# Identifying Work‐Related Factors Leading to Awkward Postures and Musculoskeletal Disorders in Crane Operators: Insights From a Grounded Theory Study

**DOI:** 10.1002/hsr2.72887

**Published:** 2026-09-23

**Authors:** Saeed Barati Chamgordani, Alireza Choobineh, Haleh Ghaem, Mahnaz Shakerian

**Affiliations:** ^1^ Student Research Committee, Department of Occupational Health and Safety Engineering, School of Health Shiraz University of Medical Sciences Shiraz Iran; ^2^ Research Center for Health Sciences, Institute of Health Shiraz University of Medical Sciences Shiraz Iran; ^3^ Department of Epidemiology, School of Health Shiraz University of Medical Sciences Shiraz Iran; ^4^ Department of Occupational Health Engineering, School of Health Isfahan University of Medical Sciences Isfahan Iran

**Keywords:** grounded theory, musculoskeletal disorders (MSDs), occupational health, awkward posture, overhead crane operators

## Abstract

**Background:**

Musculoskeletal disorders (MSDs) remain a leading cause of occupational disability worldwide. Overhead crane operators are frequently exposed to awkward postures that increase the risk of MSDs. While physical conditions are often blamed, the broader organizational and ergonomic context remains underexplored.

**Methods:**

This study employed a grounded theory approach, conducting 52 semi‐structured interviews with crane operators and safety personnel in a large Iranian steel company. Transcripts were analyzed using open, axial, and selective coding via MAXQDA software.

**Results:**

Eight interrelated categories were identified as contributing factors: individual behavior, communication practices, managerial oversight, physical infrastructure, cabin design, psychological demands, seat ergonomics, and visual limitations. The conceptual model illustrates how these systemic and personal domains converge to reinforce harmful postural patterns.

**Conclusion:**

Addressing postural risk among crane operators requires a holistic ergonomics strategy that incorporates operator feedback, redesign of work environments, and organizational reforms. The proposed model serves as a framework for targeted ergonomic interventions in high‐risk industrial settings.

## 1 Introduction

Musculoskeletal disorders (MSDs) continue to be a prominent global concern in occupational health, consistently ranking among the most frequently reported work‐related health issues across a wide range of industries [1, 2]. These disorders often manifest as pain, loss of mobility, and in severe cases, long‐term disability. The development of MSDs is closely tied to the physical demands of the job and suboptimal workplace conditions that aggravate or initiate such injuries [3]. Epidemiological studies consistently highlight the lower back, neck, and shoulders as the body regions most susceptible to WMSDs, particularly in physically intensive occupations [4, 5, 6].

Among various occupational groups, overhead crane operators are notably at high risk due to the inherent nature of their tasks [4, 7]. In heavy industrial sectors—especially steel manufacturing—overhead cranes are indispensable for material handling and workflow continuity. Operators, therefore, bear the responsibility of precisely monitoring, guiding, and maneuvering heavy loads across the workspace from enclosed cabins, often for prolonged durations. This combination of physical strain and constrained movement significantly elevates their vulnerability to musculoskeletal complaints [8]. Alarmingly, research suggests that up to 90% of crane operators experience some form of MSDs, predominantly affecting the upper and lower back, neck, and shoulders [5].

Ergonomic analysis plays a pivotal role in enhancing worker well‐being and optimizing operational efficiency by aligning workplace conditions with the physiological and psychological capacities of the workforce. While informative, many existing ergonomic evaluations often fail to capture the task‐specific nuances and real‐world complexity of crane operations [9]. Given the unique challenges presented by crane operation—ranging from prolonged postures to environmental constraints—there is a clear need for context‐sensitive ergonomic investigations. These should not only assess physical strain but also incorporate psychological, organizational, and design‐related factors that contribute to awkward postures.

To address these limitations, the current study utilizes a qualitative design to investigate the underlying factors influencing awkward posture among overhead crane operators. This approach facilitates a rich, theory‐driven understanding by integrating the lived experiences and subjective viewpoints of operators. Ultimately, the findings of this study aim to inform tailored ergonomic interventions and support safer, more sustainable practices in crane operation and other high‐risk industrial settings.

## 2 Materials and Methods

This qualitative study was conducted using the grounded theory approach to explore the underlying factors contributing to awkward posture among overhead crane operators. Semi‐structured, face‐to‐face interviews served as the primary method for data collection, offering rich insights into the lived experiences of the participants.

### 2.1 Interview Design and Study Procedure

**Figure 1:**
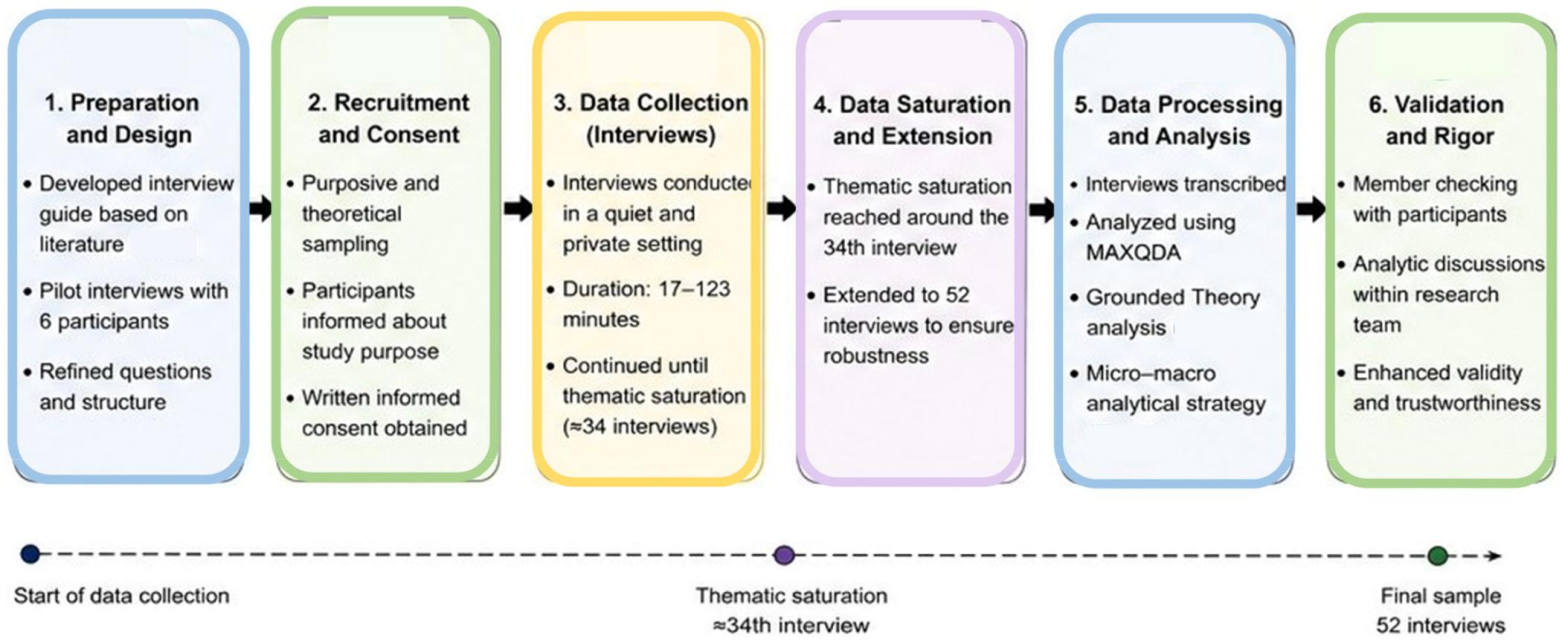
Overview of the study procedure.

An initial interview guide was developed based on relevant ergonomic and occupational health literature [10, 11], focusing on identifying root causes of awkward posture in crane operator workstations. The study was carried out in a large, long‐established steel industry complex in Iran (Figure 1).

A total of 52 participants were included: 40 crane operators, 5 team leaders, 3 mechanics foremen, 2 deputies, 1 technician, and 1 safety officer. The average age of participants was 43.5 years, with a mean work experience of 18 years. Participant selection was guided by four criteria of key informant status, peer recommendation, voluntary participation, and reference‐based sampling [12].

### 2.2 Data Analysis

**Figure 2:**
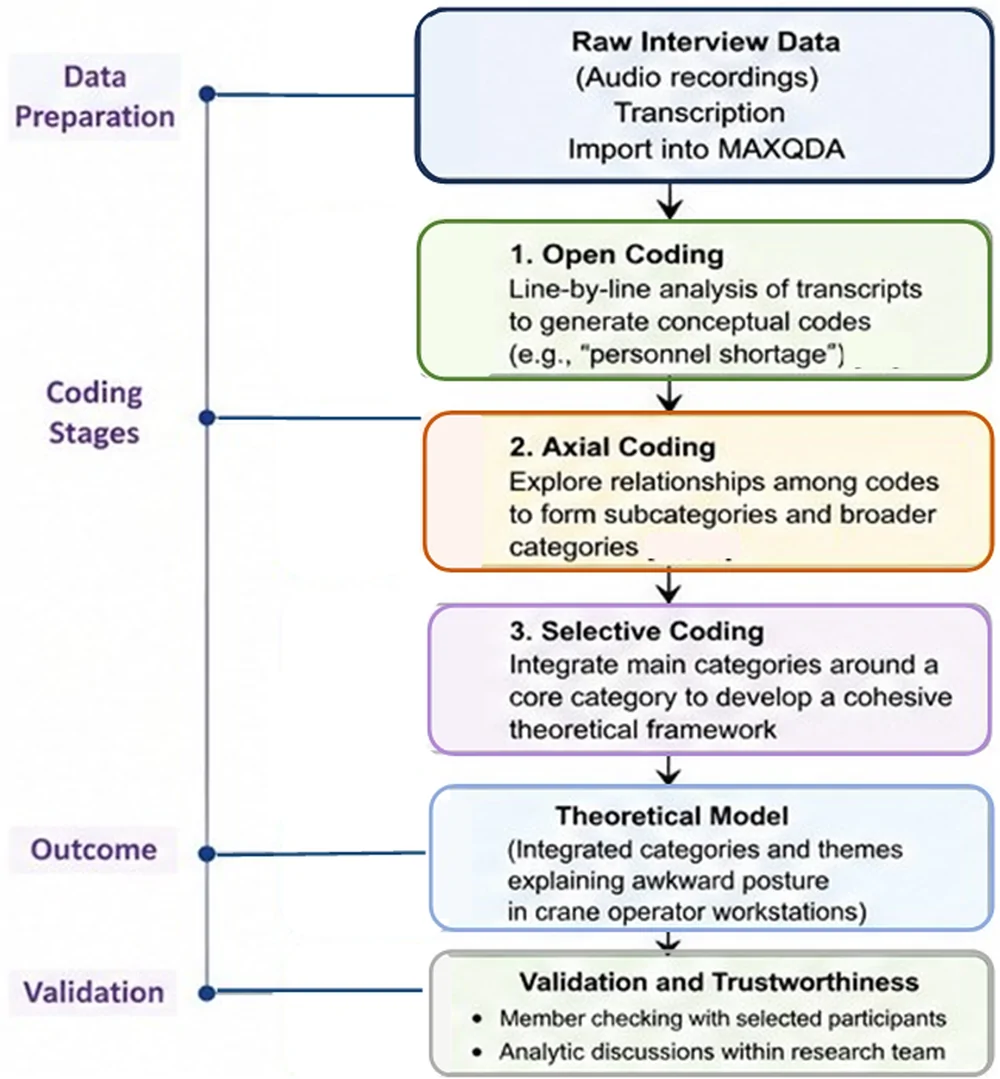
Grounded theory data analysis process.

The analysis followed a three‐stage Grounded theory coding process: open, axial, and selective coding [12]. In the open coding phase, transcripts were analyzed line by line, with segments of meaning broken down into conceptual codes [13]. During axial coding, relationships between codes were explored to form subcategories and broader categories based on conceptual interlinkage (Figure 2) [11, 14].

### 2.3 Analytical Strategy

To enhance the richness and depth of the analysis, both **micro** and **macro** approaches were applied:

- In the **micro‐level analysis**, transcripts were examined line‐by‐line. When a passage conveyed a specific idea or theme, it was selected as a unit of meaning. These units were then condensed into concise expressions reflecting the participants' viewpoints.
- In the **macro‐level analysis**, entire transcripts were treated as holistic units. Through repeated readings, subthemes were identified and grouped into higher‐order themes based on conceptual similarity [15].

**Figure 3:**
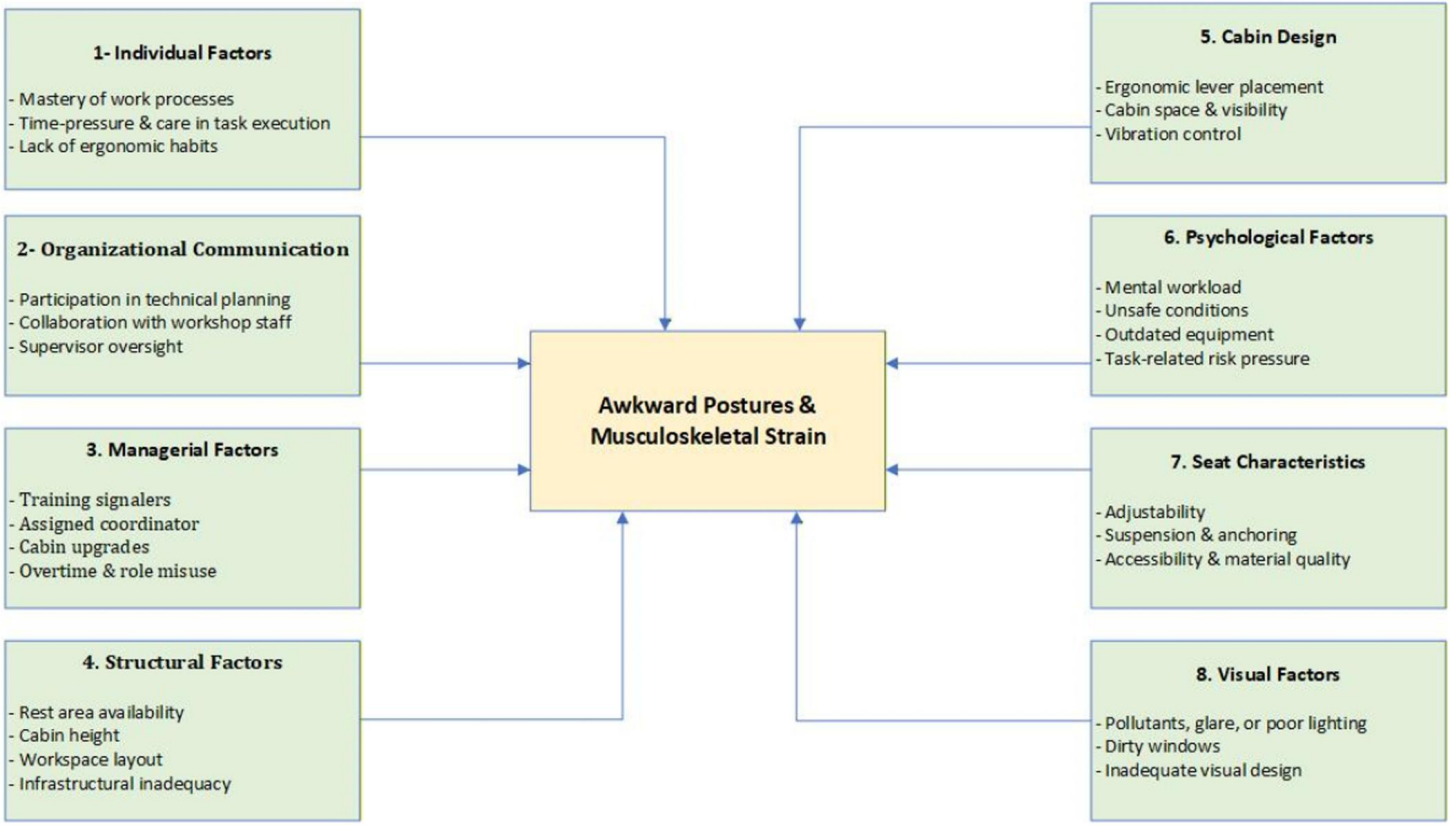
Categorization of factors influencing awkward posture among overhead crane operators.

A summary of the final categories derived from this analysis is presented in Figure 3, offering a structured overview of the factors influencing awkward posture in crane operator workstations.

## 3 Results

### 3.1 Coding Results

All interviews were analyzed, and the underlying concepts were extracted. The results reflect recurring themes and similar perspectives shared by participants, which have been integrated into a cohesive summary.

Several intermediate categories were also identified that conceptually linked raw codes to the core phenomenon. These included:

- **“Detached supervision”**—participants frequently described situations where managers were aware of ergonomic issues but remained passive or disengaged.
- **“Structural resignation”**—operators expressed a sense of resignation toward enduring discomfort, often considering poor posture a ‘normal’ aspect of crane work.
- **“Normalization of deviation”**—hazardous practices became routine, with unsafe behavior reinforced by social norms and lack of enforcement.

These intermediate categories served as conceptual bridges, helping transition from descriptive codes to a more abstract model of how awkward posture emerges and persists. The coding results are presented in Figure 3, and each category details is provided in Appendix 1.

### 3.2 Grounded Theory Conceptual Model

Axial coding revealed that awkward posture is not solely a behavioral issue but a multifactorial outcome influenced by personal, organizational, environmental, and ergonomic domains. To enrich the theoretical framework and remain consistent with grounded theory principles, the following axial coding dimensions were identified and structured:

- **Causal Conditions:** For example, lack of ergonomic training, high workload, and rushed performance to gain break time.
- **Contextual Conditions:** Such as cabin layout, workshop spatial organization, shift design, and equipment quality.
- **Intervening Conditions:** Including supervisor behavior, communication quality, and informal peer norms.
- **Action/Interaction Strategies:** Like task improvisation, physical compensation, and behavioral adaptation.
- **Consequences:** Such as chronic musculoskeletal discomfort, accumulated fatigue, and normalized awkward posture.

The proposed model integrates eight main categories—individual, communicational, managerial, structural, cabin design, psychological, seating, and visual factors—offering a holistic framework for ergonomic interventions and future research in industrial settings.

**Figure 4:**
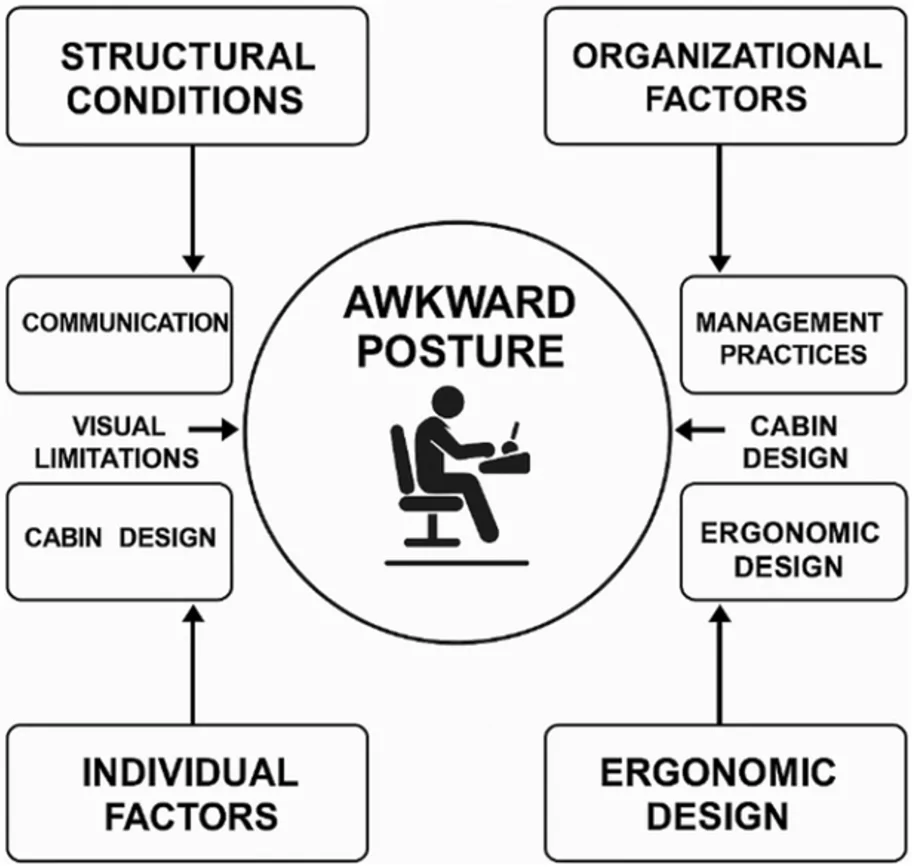
Grounded theory‐based conceptual framework of factors influencing awkward posture in crane operation.

A synthesized model of these findings is shown in Figure 4, presenting how structural limitations, visual constraints, ergonomic shortcomings, and psychosocial stressors converge to shape improper postures. It underscores the necessity for multi‐level ergonomic interventions tailored to the operational realities of crane work.

## 4 Discussion

This study aimed to uncover the underlying contributors to awkward posture among overhead crane operators using a grounded theory approach. The findings revealed eight interrelated categories shaping musculoskeletal strain: individual habits, communication practices, management, structural features, cabin design, psychological demands, seat ergonomics, and visual limitations.

### 4.1 Individual Factors

Operators' familiarity with procedures, attention to safety, and ergonomic awareness critically influence posture. Lack of training and time pressure frequently led to forward‐leaning or twisted positions. Prior studies align with these results, highlighting that time pressure and unsafe work speed can exacerbate musculoskeletal strain [16, 17].

### 4.2 Organizational Communication

Communication emerged as a crucial moderator; involving operators in equipment layout decisions and ensuring real‐time coordination during tasks significantly reduced the need for compensatory postures.

### 4.3 Managerial and Maintenance Factors

Managerial oversight and adequate staffing play protective roles. Insufficient personnel or prolonged overtime were linked to fatigue‐induced posture compensation [18]. Additionally, assigning technical personnel and ensuring timely maintenance reduced workload and supported healthier posture. Beyond these managerial responsibilities, participatory ergonomics approaches—where operators are actively engaged in identifying and resolving ergonomic challenges—have demonstrated notable effectiveness in reducing workload and musculoskeletal complaints [19].

### 4.4 Structural Conditions

The presence of rest facilities and optimally designed cabins helped alleviate these burdens, emphasizing the importance of infrastructure investment. Structural fatigue in equipment—especially in welding‐intensive crane systems—has also been linked to increased ergonomic risk [20, 21].

### 4.5 Cabin Design

The physical environment within the cabin, particularly the layout and usability of controls, was pivotal. Features like proper visibility, reduced vibration, and intuitive control design minimized strain and supported neutral postures [22].

### 4.6 Psychological Load

Psychological load from repetitive, high‐responsibility tasks influenced postural behavior—environmental stressors—noise, insufficient lighting, and communication disruptions—amplified tension and diminished ergonomic awareness. Stressful environments led operators to overlook posture, especially under time pressure [21].

### 4.7 Operator Seat Characteristics

Seat quality, adjustability, and material composition impacted comfort and musculoskeletal resilience. Features like adjustable height, lumbar support, and shock absorption helped reduce injury risk [21, 23, 24].

### 4.8 Visual Factors

Visual obstructions from dirty windows, glare, or poor lighting conditions forced operators to adjust their posture awkwardly. Prior research confirms that inadequate visual environments increase strain on the neck and upper back [23, 25].

### 4.9 Model Integration and Implications

These interconnected themes are visualized in a conceptual framework illustrating how systemic, personal, and environmental factors converge to influence posture (Figure 4). It explains the coherence among all extracted categories and emphasizes how awkward postures endure not because of isolated failures, but due to a broader, systemic quietude that resists adaptive change [26, 27, 28, 29, 30]. Adopting a systems‐level view may be critical to reducing the high prevalence of work‐related musculoskeletal disorders in the steel industry and similar sectors.

## 5 Conclusion

Awkward posture among overhead crane operators is influenced by a combination of ergonomic, organizational, structural, and psychological factors. Key contributors identified in this study included poor cabin design, inadequate visibility, high workload, insufficient staffing, weak communication, and lack of ergonomic support. The grounded theory model developed in this study provides a practical framework for identifying ergonomic risks and designing targeted interventions. Improvements in seating, cabin layout, visibility, operator participation, and management practices may reduce musculoskeletal strain and enhance operational safety. Future research should evaluate these factors quantitatively across larger industrial populations to support evidence‐based ergonomic interventions.

## Author Contributions

**Saeed Barati Chamgordani:** methodology, software, formal analysis, visualization, resources, investigation, data curation, writing – original draft, writing – review and editing, funding acquisition. **Alireza Choobineh:** conceptualization, supervision, project administration, funding acquisition. **Haleh Ghaem:** formal analysis. **Mahnaz Shakerian:** conceptualization, methodology, formal analysis, writing – review and editing, project administration, validation.

## Ethics Statement

This study was approved by the Ethics Committee of Shiraz University of Medical Sciences (Ethics Code: IR. SUMS. SCHEANUT. REC.1403.010). All participants were fully informed about the study's objectives and procedures, and written informed consent was obtained from each participant prior to their involvement. The study was conducted in accordance with the Declaration of Helsinki.

## Conflicts of Interest

The authors declare no conflicts of interest.

## Transparency Statement

The corresponding author, Mahnaz Shakerian, affirms that this manuscript is an honest, accurate, and transparent account of the study being reported; that no important aspects of the study have been omitted; and that any discrepancies from the study as planned (and, if relevant, registered) have been explained.

## Data Availability

The data that support the findings of this study are available from the corresponding author upon reasonable request.

## Appendix 1

See Table A1.

**Table A1:** The details regarding the categorization of factors contributing to awkward posture organized into eight main domains based on axial coding.

*1. Individual Factors* **Personal habits and operational behavior** emerged as key contributors to awkward postures among crane operators. Participants emphasized the necessity of mastering workshop‐specific workflows, noting that even experienced operators experience stress when unfamiliar with a new environment, often resulting in compensatory leaning or twisting to maintain control. Additionally, many operators admitted to rushing through tasks to maximize rest time, often sacrificing ergonomic posture in the process. Lack of adherence to basic ergonomic principles during work and leisure—such as proper sitting, movement, and rest habits—further compounds musculoskeletal strain. These behaviors suggest that posture‐related risks are not limited to the work setting but extend to broader lifestyle patterns that shape bodily stress responses over time. *2. Organizational Communication* **Workplace interactions** significantly shape the ergonomic safety of crane operators. When operators were involved in technical decision‐making—such as layout changes or equipment upgrades—the resulting modifications were more efficient and less physically taxing. In contrast, a lack of consultation often led to operational inefficiencies and unnecessary crane maneuvering. Collaboration between floor staff and crane operators was also critical. Participants reported that helpful behaviors—such as clear signaling, avoiding interference with load paths, and maintaining calm under pressure—reduced their mental load and allowed for healthier posture during operation. Conversely, miscommunication and a lack of supervisory oversight increased physical and psychological strain, frequently forcing operators into awkward body positions to compensate for others’ errors or to gain better visibility and control. *3. Managerial Factors* Managerial shortcomings, such as lack of trained signalers, absence of dedicated coordinators for operator issues, and inability to upgrade outdated cabins, were found to exacerbate awkward postures. Mandatory overtime and staff shortages increase operator fatigue, while the lack of ergonomic training contributes to poor posture habits. Misuse of operator roles in organizational charts further amplifies workload and stress. *4. Structural Factors* Environmental conditions—such as inadequate rest areas, improper cabin height, obstructed crane paths, and poor work area positioning—significantly influence posture. Operators exposed to poorly adapted cranes and growing production demands without infrastructure upgrades are more likely to adopt harmful static postures. *5. Cabin Design* Outdated cabin designs with limited space, suboptimal lever placement, poor vibration control, and obstructed visibility contribute to musculoskeletal strain. In contrast, modern ergonomic layouts improve posture by enhancing accessibility, visual control, and communication with ground personnel. *6. Psychological Factors* High workload, responsibility, and fear of accidents place psychological pressure on operators. Unsafe working conditions, defective equipment, and the need for extreme vigilance cause operators to unconsciously adopt poor posture for prolonged periods. *7. Operator Seat Suitability* Seat features such as adjustable height and backrest, vibration‐dampening suspension, proper anchoring, and ergonomic positioning significantly affect posture. Inaccessible or poorly constructed seats increase the risk of musculoskeletal issues. *8. Visual Factors* Visual barriers caused by pollutants, dirty windows, glare, or inadequate lighting force operators into awkward postures. Ensuring clear visibility reduces physical strain and enhances safety.
